# Hedgehog Components Are Present in Polymorphous Adenocarcinoma of the Salivary Gland Regardless of *PRKD1* Mutation and Tissue Invasion

**DOI:** 10.1111/jop.70057

**Published:** 2025-09-10

**Authors:** Dandara Andrade De Santana, Cecília Vitória Lima De Oliveira, Flávia Caló De Aquino Xavier, Manoela Domingues Martins, Bruno Cunha Pires, Tercio Guimarães Reis, Patrícia Ramos Cury, Clarissa Araújo Gurgel, Maria Cristina Teixeira Cangussu, Daniel Araki Ribeiro, Victor Coutinho Bastos, Carolina Cavalieri Gomes, Jean Nunes Dos Santos

**Affiliations:** ^1^ Postgraduate Program in Dentistry and Health, School of Dentistry Universidade Federal da Bahia Salvador Bahia Brazil; ^2^ School of Dentistry Federal University of Bahia Salvador Brazil; ^3^ Laboratory of Oral and Maxillofacial Pathology, School of Dentistry Universidade Federal da Bahia Salvador Bahia Brazil; ^4^ School of Dentistry, Department of Oral Pathology Universidade Federal do Rio Grande do Sul – UFRGS Porto Alegre Rio Grande do Sul Brazil; ^5^ Centro de Diagnóstico Pires Feira de Santana Bahia Brazil; ^6^ Hospital Otorrinos Feira de Santana Bahia Brazil; ^7^ Department of Periodontology Universidade Federal da Bahia Salvador Bahia Brazil; ^8^ Department of Bioscience Institute of Health and Society, Universidade Federal de São Paulo São Paulo Brazil; ^9^ Department of Pathology Biological Science Institute, Universidade Federal de Minas Gerais Belo Horizonte Brazil

**Keywords:** adenocarcinoma, Hedgehog proteins, mutation, salivary gland neoplasms

## Abstract

**Purpose:**

Polymorphous adenocarcinoma of the salivary gland is characterized by cellular uniformity associated with a variety of morphological growth patterns, a fact that makes its diagnosis challenging. Therefore, the identification of genetic alterations and signaling pathways emerges as a tool for elucidation of the pathogenesis of this tumor and accurate differential diagnosis. The aim of this study was to assess mutations in the *PRKD1* gene and in protein components of the HH pathway (SHH, IHH, SMO, and GLI‐1) in cases of polymorphous adenocarcinoma of the salivary gland.

**Methods:**

Sanger sequencing was used to interrogate hotspot mutations in *PRKD1* exon 15 and immunohistochemistry to analyze the protein expression of PRKD1, SHH, IHH, SMO, and GLI‐1.

**Results:**

The *PRKD1* c.2130A>C/T hotspot mutation was detected in 50% of the sequenced samples. A previously unreported variant, c.2110C>T resulting in p.His704Tyr, was identified in one case, while 100% of the samples carried the intronic variation rs2273813, regardless of tissue invasion (perineural, lymphovascular, fat, bone, muscle, and mucous acini). Immunostaining revealed significant results for several associations between PRKD1, IHH, SMO, and GLI‐1. In contrast, SHH immunoexpression did not correlate with the expression of the other proteins.

**Conclusion:**

The *PRKD1* E710D hotspot mutation and protein expression of PRKD1 and HH components (SHH, IHH, SMO, and GLI‐1) were detected in PAC regardless of tissue invasion, although HH proteins contributed to the morphogenesis of this rare oral cancer. Additionally, the positive correlation between the expression of PRKD1 and HH pathway components (IHH, SMO, and GLI‐1) suggests a possible interaction between these proteins, independent of the HH pathway ligand.

## 1 Introduction

Salivary gland tumors are a group of tumors whose biological behavior ranges from indolent to aggressive. Significant morbidity and mortality are observed for some malignant neoplasms [1]. According to the most recent World Health Organization (WHO) classification for head and neck tumors, salivary gland tumors comprise a group of 36 distinct histopathological types, including the polymorphous adenocarcinoma (PAC). It is a rare neoplasm, which is characterized by cytological uniformity, morphological variety, and an infiltrative growth pattern [2].

In general, PAC of the salivary glands has been associated with alterations in the protein kinase D (*PRKD*) gene family, including rearrangements in *PRKD1/2/3* and the *PRKD1* p.Glu710Asp (E710D) hotspot mutation [2]. The E710D mutation in the *PRKD1* gene contributes to tumor progression by increasing kinase activity, favoring cell proliferation. Thus, this mutation has been recognized as a relevant diagnostic finding and as a therapeutic target for PAC of the salivary gland [3, 4, 5, 6].

Protein kinase D1 (PRKD1) is a serine/threonine kinase that exerts key functions in several signaling pathways and many cellular processes, including cell migration, differentiation, survival, and adhesion [7, 8, 9]. Due to its important role in cellular metabolism, this kinase plays diverse roles in promoting or suppressing tumorigenesis and metastasis, particularly in solid tumors [10]. Likewise, protein kinases can influence the Hedgehog (HH) pathway through the phosphorylation of proteins associated with this signaling cascade, including the Smoothened receptor (SMO) and the three transcription factors of the glioma‐associated oncogene (GLI) family (GLI‐1, GLI‐2, and GLI‐3).

The HH pathway plays an essential role in embryonic development and in the maintenance of healthy tissues, participating in the regulation of stem cells in normal tissues [11]. On the other hand, the deregulation or aberrant activation of the HH pathway is associated with tumor development and progression, including the regulation of epithelial–mesenchymal transition [12, 13]. Its components play key roles in the regulation of genes related to cell proliferation, cell survival, invasion, and angiogenesis [11, 14].

The Sonic Hedgehog pathway is activated by the homologous proteins Sonic Hedgehog (SHH) and Indian Hedgehog (IHH), which exert similar functions mainly related to tissue development, tissue homeostasis, and tumorigenesis [14, 15]. Both proteins bind to the transmembrane receptor Patched1 (PTCH1), thereby blocking its inhibitory function on the SMO receptor. The activation of SMO triggers an intracellular cascade that leads to the activation of GLI family transcription factors, especially GLI‐1. In the nucleus, GLI‐1 regulates the expression of target genes involved in the control of cell proliferation, survival, invasion, and angiogenesis, such as *CCND1*, *BCL2*, *MMP9*, *MYC, SOX2*, and *ZEB1* [16, 17].

To our knowledge, there are no data on the participation of the HH pathway in PAC of the salivary gland or in cases carrying somatic mutations, although previous authors suggested activation of the HH pathway in pleomorphic adenoma, adenoid cystic carcinoma, and mucoepidermoid carcinoma [18]. Therefore, the aim of this study is to interrogate mutations in the *PRKD1* gene and in protein components of the HH pathway (SHH, IHH, SMO, and GLI‐1) in PAC of the salivary gland. It is important to note that identifying somatic mutations and new protein markers would provide valuable diagnostic tools and strategically efficient therapeutic targets.

## 2 Materials and Methods

### 2.1 Sample Selection

After approval of the study by the Ethics Committee of the School of Dentistry, Federal University of Bahia (FOUFBA; Protocol number 58095321.3.0000.5024), a convenience sample composed of 19 cases of PAC of the salivary gland was included. All cases were retrieved from the databases of the Pathology Laboratory of FOUFBA.

Formalin‐fixed, paraffin‐embedded (FFPE) surgical specimens with preserved tissues suitable for microscopic analysis were selected. Cases without biologically preserved tissue, paraffin blocks containing little material, cases with unavailable and/or incomplete clinical data, and cases with insufficient DNA for analysis were excluded from the study. Patient records and biopsy forms were used as original sources for the collection of clinicopathological data.

### 2.2 Histopathological Study

Fifteen cases were selected, and new histological slides were stained with hematoxylin/eosin. The diagnosis of salivary gland PAC was confirmed by an experienced oral pathologist considering the criteria recommended by the WHO [2]. The cases were then classified into the classic, cribriform, papillary, and indeterminate types [19]. Indeterminate tumors exhibited classic and cribriform features, while tumors with ≥ 50% of a papillary architecture were classified as papillary. The presence of perineural and lymphovascular invasion and infiltration of fat, bone, muscle, and mucous acini was also evaluated.

### 2.3 DNA Extraction

Genomic DNA was extracted using the QIAamp DNA FFPE Tissue Kit (Qiagen, Germany) according to the manufacturer's instructions. The purity and concentration of the genomic DNA were determined in a NanoDropIM spectrophotometer (Thermo Fisher Scientific, USA) and the OD 260/280 ratio ranged from 1.8 to 2.0.

### 2.4 PCR Amplification

The *PRKD1* gene was analyzed by standard PCR amplification in eight salivary gland PAC samples. The PCR assays were performed using MyTaq HS Red Mix (2× Bioline Reagents, UK) and M13‐tailed primers to amplify a 210‐bp fragment of *PRKD1* exon 15 encompassing the p.E710D (c.2130A>T or c.2130A>C) hotspot mutation. The following M13‐tailed primers were used: forward 5′‐GTAAAACGACGGCCAGTcagATACTCGTGGCTTTGCG‐3′ and reverse 5′‐CAGGAAACAGCTATGACaggtgacaagatgctacatgga‐3′.

Negative and positive controls were included in all PCR assays. PCR products were purified using ExoSAP‐IT PCR Product Cleanup Reagent (Thermo Fisher Scientific).

### 2.5 Sanger Sequencing

The purified PCR products were bidirectionally sequenced using the Big Dye Terminator v3.1 Cycle Sequencing Kit (Applied Biosystems, USA) on an ABI 3730 DNA Analyzer (Applied Biosystems). The chromatograms were manually inspected in the Snap Gene Viewer program using the comparison sequence.

### 2.6 *In Silico* Analysis

Computational tools were used to identify pathogenicity predictors reported in bioinformatics databases for *PRKD1* mutations detected in samples of salivary gland PAC. The following tools were used for computational prediction: SIFT (Sorting Intolerant From Tolerant) available at https://sift.bii.a‐star.edu.sg; PolyPhen‐2 (Polymorphism Phenotyping v2) available at http://genetics.bwh.harvard.edu/pph2, and MutationTaster available at https://www.mutationtaster.org. Additionally, we used the UMD‐Predictor Pro tool of the Genomnis platform (available at https://umd‐predictor.genomnis.com), which permits mutation analysis to predict pathogenicity scores in missense and synonymous single nucleotide variants.

Single nucleotide polymorphisms (SNPs) found in the cases of salivary gland PAC were correlated with gene expression levels in normal salivary gland tissues using the Genotype‐Tissue Expression (GTEx) Portal at https://www.gtexportal.org. Mapping expression quantitative trait loci (eQTL) permits us to determine whether the SNP of interest influences the expression of specific genes, as well as the magnitude of this effect [20].

### 2.7 Immunohistochemistry

The specimens were cut into 3‐μm‐thick sections and mounted on silanized glass slides. The Advance System (Dako Corporation, USA) was applied according to the manufacturer's protocol. Endogenous peroxidase was blocked using a 3% hydrogen peroxide solution for 10 min (2×), followed by antigen retrieval with EDTA in a water bath for 20 min. The slides were then incubated overnight at 4°C with the primary antibodies diluted in a background reduction solution (Dako Corporation, Carpinteria, USA), as described in Table S1.

The reactions were developed with 3,3′‐diaminobenzidine (DAB; Dako Corporation, Carpinteria, USA) and counterstained with Harris hematoxylin. For immunohistochemical analysis, two independent examiners scored the cases based on the intensity and proportion of staining using the criteria described in previous studies [18, 21]. The distribution of staining between cytoplasm, nucleus, and membrane was also analyzed under a high‐definition microscope (AxiostarPlus, Zeiss, Germany, 2008).

### 2.8 Statistical Analysis

The data were analyzed using IBM SPSS Statistics for Windows, version 29.0.2 (Armonk, NY, USA: IBM Corp.). The chi‐square test was applied to evaluate the association between PRKD1 and the protein markers of the HH pathway. Pearson correlation was used to examine the linear relationship in the quantitative expression of these protein markers. In addition, scatter plots were generated with the Jamovi software (version 2.3) to explore the relationships between continuous variables. The level of significance was set at *p* < 0.05.

## 3 Results

### 3.1 Sample

The sample consisted of 15 cases of salivary gland PAC (Figure S1) represented by swelling or nodules. Available clinical and demographic data indicated that 71% (*n* = 10) were females and 29% (*n* = 4) were males, with a mean age of 59.5 years (range 34 to 85 years ±10.85). Eight cases were located in the palate, whereas seven cases occurred in the upper lip, and only two cases occurred in the buccal mucosa (Figure S1).

Perineural invasion was present in four cases (26.7%) and lymphovascular invasion in one (6.7%). Infiltration of fat (*n* = 5, 33.3%), bone (*n* = 3, 20%), striated muscle (*n* = 2, 13.3%), and mucous acini (*n* = 4, 26.7%) was also observed.

### 3.2 Analysis of *PRKD1* Mutations

PCR amplification failed in 7 cases, resulting in 8 sequenced cases. The *PRKD1* c.2130A>C/T hotspot mutation was detected in 50% (4/8) of the cases studied (Figure S2).

Morphological variation was found in the four PAC cases carrying the E710D hotspot mutation, with 25% (1/4) of the cases being classified as classic, 25% (1/4) as cribriform, and 50% (2/4) as indeterminate.

**FIGURE 1:**
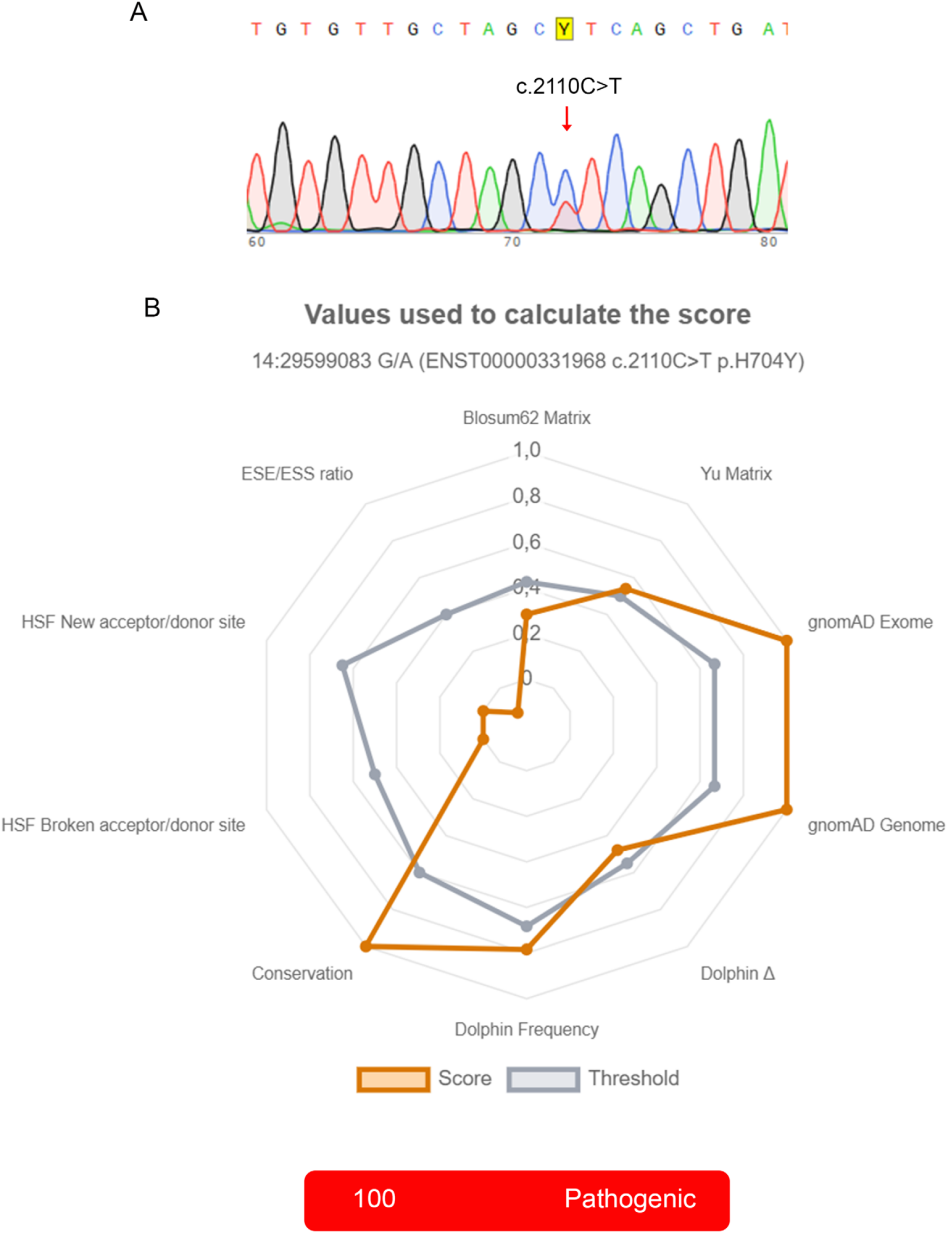
(A) Chromatogram of the variant detected at codon 704 of the *PRKD1* gene (c.2110C>T; p.His704Tyr; p.H704Y). (B) *In silico* pathogenicity analysis using the UMD‐Predictor Pro tool of the Genomnis platform.

Case 11 simultaneously carried the *PRKD1* c.2130A>C hotspot mutation and a new variant at codon 704 in exon 15 of the *PRKD1* gene (c.2110C>T; p.His704Tyr; p.H704Y), which has not yet been reported in the literature (Figure 1A). *In silico* pathogenicity analysis using the UMD‐Predictor Pro tool of the Genomnis platform assigned a maximum score of 100 to the new mutation, indicating its high pathogenic potential (Figure 1B).

**FIGURE 2:**
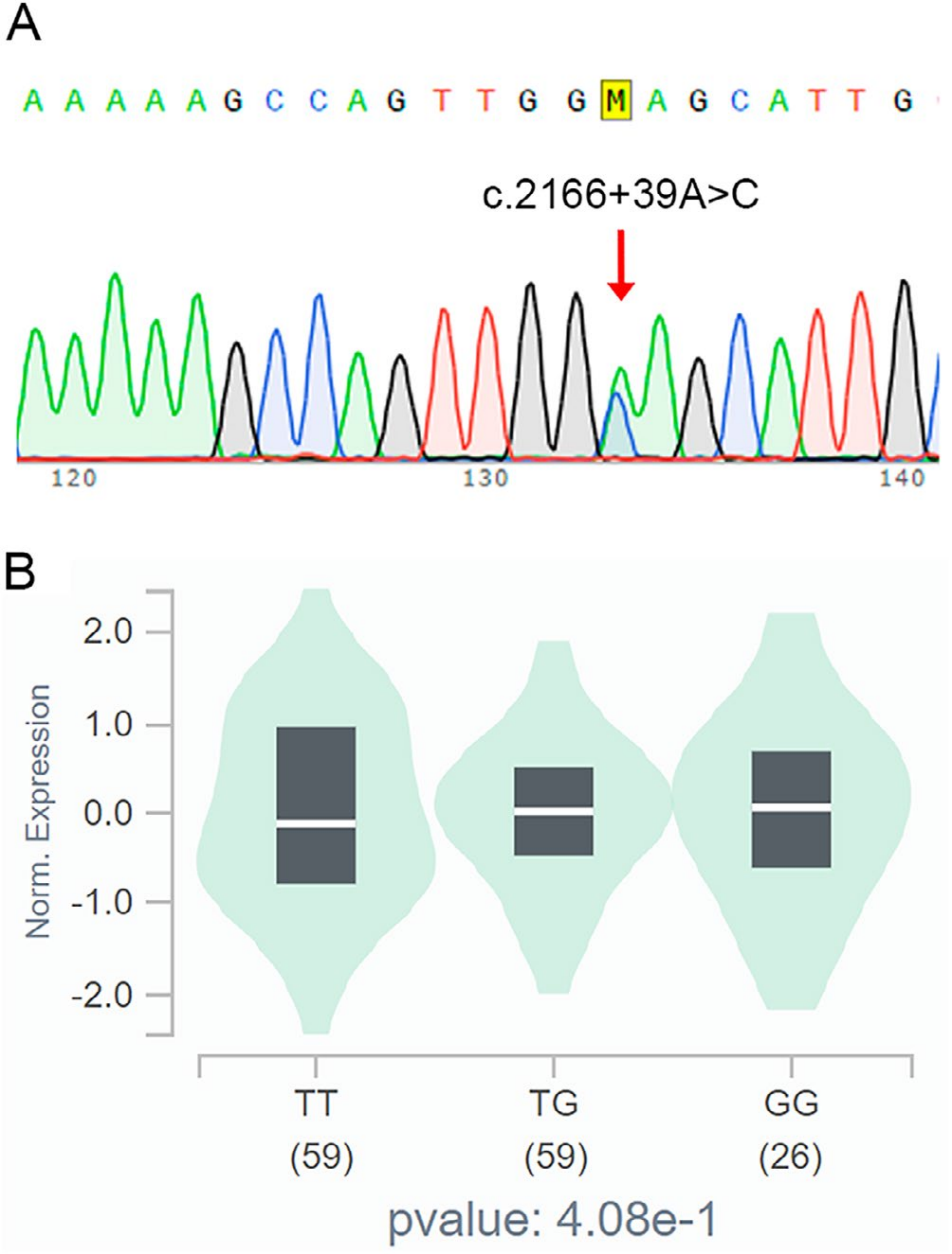
(A) Chromatogram of the rs2273813 intronic variation. (B) Correlation between the rs2273813 SNP and gene expression levels in normal salivary gland tissues using the Genotype‐Tissue Expression (GTEx) platform.

Additionally, we detected the rs2273813 SNP, an intronic variation of the *PRKD1* gene, in all samples (Figure 2A). The SNP identified in all cases has no influence on the expression of the *PRKD1* gene, that is, there is no significant difference in the expression of the *PRKD1* gene in normal minor salivary glands between individuals who do not carry the rs2273813 SNP and individuals who are homozygous or heterozygous carriers of this polymorphism (Figure 2B).

Table S2 shows a synthesis of the results of studies that investigated the *PRKD1* E710D mutation in PAC, including the number of cases analyzed, distribution by gender and age, the method used for detection of the mutation, and the histological patterns observed in mutated cases. The frequency of the mutation ranged from 11.1% to 72.9%, including the result of the present study (50%).

### 3.3 Immunohistochemical Analysis

Table S3 summarizes the distribution of immunostaining of the proteins studied in the cases of PAC. The PRKD1 protein was mainly expressed at low levels (*n* = 11, 73.3%), while high expression was observed in 3 (20.0%) cases. Regarding HH pathway proteins, low expression of IHH was found in 8 (53.3%) cases and high expression in 6 (40.0%). SMO and GLI‐1 were mainly expressed at high levels (*n* = 14, 93.3%). The same was found for SHH, which exhibited high expression in 13 (86.7%) cases. Expression of PRKD1, IHH, and GLI‐1 was absent in only one case.

**FIGURE 3:**
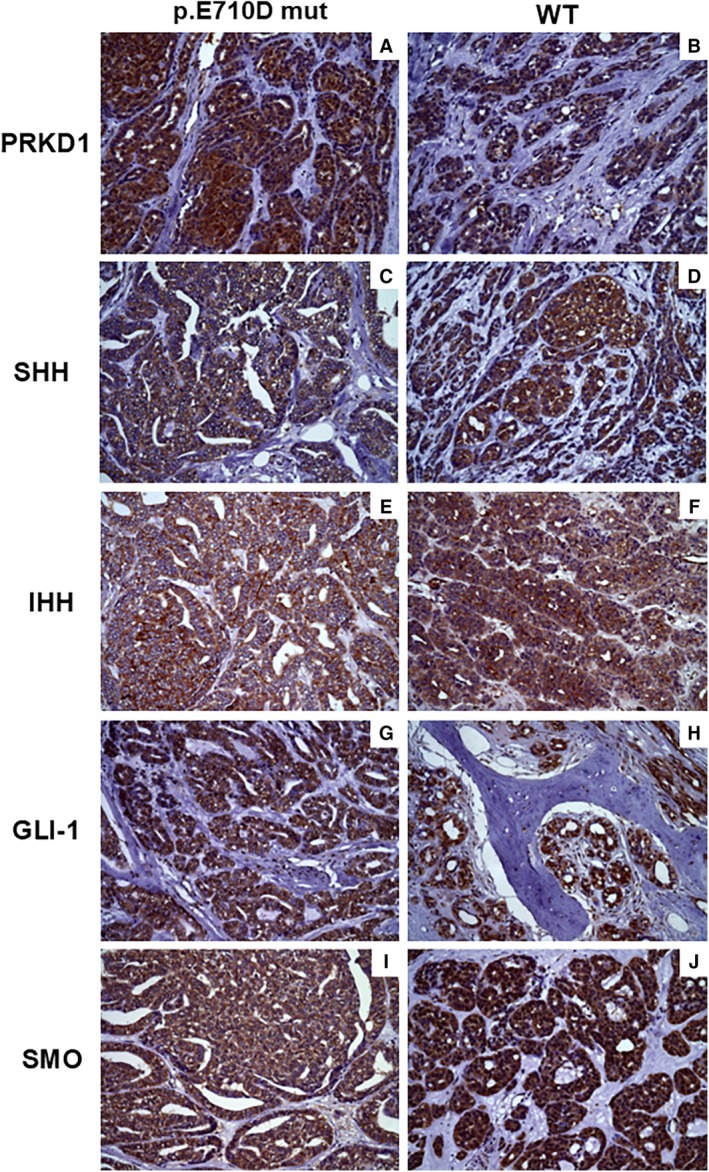
Immunostaining of HH pathway proteins and PRKD1 in cases of polymorphous adenocarcinoma carrying the E710D mutation and wild‐type cases.

PRKD1 and SHH were present in the cytoplasm in most cases. Proteins IHH, SMO, and GLI‐1 showed a more diverse distribution between the cytoplasmic, nuclear, and membrane compartments (Figure 3). Stromal fibroblasts were positive for all proteins studied in a small number of samples.

Individual analysis of each case considering the presence or absence of the mutation and staining index of HH pathway proteins revealed high staining indices for SHH in all cases, regardless of the presence of the mutation. The same was observed for SMO and GLI‐1, except for case 8 with absent and low staining, respectively. PRKD1 exhibited a low staining index in most cases that did not carry the mutation (*n* = 3). In the mutated cases, staining for IHH was absent and low in 50% (2/4) of the cases (Table 1).

**TABLE 1:** Relationship between the E710D mutation in the *PRKD1* gene and immunohistochemical staining index of Hedgehog pathway proteins regarding those cases with PCR amplification.

Case E710D		Classification		Staining index				
		Xu et al. (2016)	WHO (2022)	PRKD1	SHH	IHH	SMO	GLI‐1
3	No	Indeterminate	Conventional	Low	High	Low	High	High
4	Yes	Indeterminate	Conventional	High	High	Low	High	High
8	Yes	Classic	Conventional	Absent	High	Absent	Low	Absent
9	No	Classic	Conventional	Low	High	High	High	High
11	Yes	Cribriform	Cribriform	Low	High	High	High	High
12	No	Classic	Conventional	High	High	High	High	High
13	Yes	Indeterminate	Conventional	High	High	High	High	High
14	No	Indeterminate	Conventional	Low	High	Low	High	High

The chi‐square test identified significant associations between PRKD1, IHH, SMO, and GLI‐1, while SHH was not significantly associated with any of the proteins (Table 2). There was no statistically significant difference in any of the immunomarkers studied between cases with the E710D mutation and wild‐type cases.

**TABLE 2:** Association matrix and statistical significance between immunostaining for HH pathway proteins and PRKD1.

		PRKD1	SHH	IHH	SMO
SHH	Chi‐square test	0.839	—		
	*p*	0.657	—		
IHH	Chi‐square test	15.9	2.02	—	
	*p*	0.003**	0.364	—	
SMO	Chi‐square test	15.0	0.165	15.0	—
	*p*	< 0.001***	0.685	< 0.001***	—
GLI‐1	Chi‐square test	15.0	0.165	15.0	15.0
	*p*	< 0.001***	0.685	< 0.001***	< 0.001***

*Note*: *p < 0.05, **p < 0.01, ***p < 0.001.

Pearson's correlation matrix plot illustrates the strength and direction of the associations shown in Figure S3. The data revealed a significant positive correlation between PRKD1 and IHH (*r* = 0.523, *p* = 0.045), SMO (*r* = 0.607, *p* = 0.016), and GLI‐1 (*r* = 0.607, *p* = 0.016). The expression of IHH was also positively correlated with both SMO (*r* = 0.598, *p* = 0.019) and GLI‐1 (*r* = 0.598, *p* = 0.019). Protein SMO was positively correlated with GLI‐1 (*r* = 1.00, *p* = 0.001), while SHH showed weak or negative correlations with the other proteins (Figure S3). The presence of the E710D mutation or immunostaining for the proteins studied did not show a statistically significant association with tumor invasion or with the classification or subtypes of PAC (Table 1).

## 4 Discussion

The results of this study revealed the presence of heterozygous somatic variants in the *PRKD1* gene, specifically c.2130A>T and c.2130A>C, in 50% (4/8) of the sequenced cases of salivary gland PAC; both variants result in a p.Glu710Asp amino acid substitution (E710D). This finding is consistent with previous studies that reported a similar frequency of mutations ranging from 50% to 56% in PAC (Table S2) [3, 4, 22].

The analysis of mutations is therefore essential in order to understand their impact and the mechanisms of associated diseases [23, 24] *PRKD1* p.Glu7Asp can mimic a phosphorylated form of the protein, maintaining it in a constitutively active state that favors protumor signals. Thus, the increased activity and overexpression of the *PRKD1* gene in PAC are the result of the gain‐of‐function E710D hotspot mutation [3, 5, 25, 26, 27].

In the present study, analysis of the expression of PRKD1 and HH pathway proteins (SHH, IHH, SMO, and GLI‐1) revealed a similar distribution and cellular localization of PRKD1 and SHH, with a predominance in the cytoplasm, while IHH, SMO, and GLI‐1 exhibited a more varied distribution. These findings might be attributed to autocrine and paracrine mechanisms as observed in other cancers [28]. Furthermore, we found high nuclear expression particularly of GLI‐1, which is one of the target genes of the HH pathway. There was also a positive correlation between GLI‐1/IHH. These findings suggest activation of the pathway, considering that IHH is homologous to SHH, and support the participation of components of the HH pathway in the development of PAC. Other salivary gland tumors, such as pleomorphic adenoma, adenoid cystic carcinoma, and mucoepidermoid carcinoma, are characterized by strictly cytoplasmic and nuclear expression of SHH and GLI‐1, respectively [18]. Similarly, the predominantly nuclear expression of protein GLI‐1 was also found in other salivary gland neoplasms, including basal cell adenocarcinoma [29]. Taken together, these localization patterns reflect the dynamics of HH pathway signaling and its implications for tumor biology and morphogenesis.

Individual analysis of the sequenced cases revealed variations in the expression of PRKD1 and IHH, suggesting an association of the E710D mutation with specific alterations in the expression or regulation of the two proteins. In contrast, SHH, SMO, and GLI‐1 exhibited more uniform expression patterns, irrespective of the presence of the mutation. These findings suggest that the mutation may modulate specific targets of the HH pathway without compromising the activation of genes that promote neoplastic proliferation.

Another important consideration is the fact that PRKD1 can influence the activation or repression of other components of the HH pathway. Similarly, mutations may also be identified in other exons or codons of the *PRKD1* gene; for example, we detected a mutation in exon 15 at codon 704 (c.2110C>T; p.His704Tyr; p.H704Y) of a cribriform PAC; to our knowledge, this mutation has not yet been reported in the literature. In this context, the use of next‐generation sequencing technologies may offer greater sensitivity and coverage, allowing the identification of additional variants or low‐frequency mutations that were undetectable with Sanger sequencing. In addition, functional studies using in vitro or in vivo models would be valuable to elucidate the precise molecular consequences of E710D and H704Y mutations in cellular signaling and tumor progression.

Our data revealed a significant positive correlation between PRKD1 and HH pathway proteins. Elevated expression of PRKD1 is associated with a concomitant increase in the expression of IHH, SMO, and GLI‐1, although the SHH binding protein showed weak or negative correlations with the other proteins studied. In addition, positive correlations were observed between IHH and GLI‐1 and between SMO and GLI‐1. Variations in the expression of SMO and GLI‐1 are directly related since SMO regulates the processing and activation of GLI‐1 in the HH pathway [14, 17]. These results reinforce the hypothesis of functional interaction between PRKD1 and HH signaling components, possibly contributing to PAC pathogenesis through synergistic oncogenic pathways.

Regarding tissue invasion (perineural, lymphovascular, fat, bone, muscle, and mucous acini) observed in 13 cases (86.7%), we found no association with the presence of the mutation or immunostaining of the proteins analyzed. Similarly, there was no association between the proteins studied and the presence of the mutation in the *PRKD1* gene. These findings suggest that protein expression occurred independently of the presence of the mutation, tissue invasion, and histopathological pattern (classic, cribriform, papillary, and indeterminate) in the PAC cases studied.

The limitations of our study include the insufficient amount and quality of the genetic material in seven cases, which reduced the total number of samples for Sanger sequencing; however, we must consider that salivary gland PAC is a rare malignant neoplasm. Further studies using other methodologies may be able to elucidate molecular drivers of this neoplasm, especially for cases carrying wild‐type *PRKD1*. However, salivary gland PAC lacking *PRKD1* somatic mutations or *PRKD* gene family rearrangements is unlikely to harbor somatic mutations involving the *PRKD2* or *PRKD3* gene [30].

## 5 Conclusions

The *PRKD1* p.Glu7Asp hotspot mutation was detected in 4/8 cases (50%), and PRKD1 protein expression was observed in 93.3% of cases. These findings are relevant for the diagnosis of PAC of the salivary gland. However, they occurred independently of tissue invasion (perineural, lymphovascular, fat, bone, muscle, and mucous acini) and proteins related to the HH pathway (SHH, IHH, SMO, and GLI‐1), which contributed to the morphogenesis of this rare cancer. Additionally, the positive correlation between PRKD1 and HH pathway components (IHH, SMO, and GLI‐1) suggests a possible interaction between these proteins, independent of the SHH ligand. Taken together, these data provide insights into the E710D hotspot mutation and HH components in PAC.

## Author Contributions

**D.A.D.S.:** methodology, validation, investigation, formal analysis, data curation, writing and review (original draft), visualization. **C.V.L.D.O.:** methodology, validation, investigation, formal analysis, data curation, review (original draft), visualization. **F.C.D.A.X.:** conceptualization, methodology, writing (review and editing), visualization. **M.D.M.:** validation, data curation, writing (review and editing), funding acquisition. **B.C.P.:** validation, methodology, data curation, writing (review and editing). **T.G.R.:** methodology, investigation, data curation, writing (review and editing). **P.R.C:** visualization, validation, formal analysis, writing (review and editing). **C.A.G.:** visualization, validation, investigation, data curation, writing (review and editing). **M.C.T.C.:** visualization, validation, formal analysis, writing (review and editing). **D.A.R.:** methodology, investigation formal analysis, writing (review and editing). **V.C.B.:** methodology, investigation, formal analysis, writing (review and editing). **C.C.G.:** visualization, validation, methodology, investigation, formal analysis, writing (review and editing). **J.N.D.S.:** conceptualization, methodology, investigation, formal analysis, writing (original draft), writing (review and editing), supervision, project administration, funding acquisition.

## Conflicts of Interest

The authors declare no conflicts of interest.

## Supporting information


**Figure S1:** Clinicopathological and demographic data and mutations of polymorphous adenocarcinomas of the salivary gland.

**Figure S2:** Sanger sequencing chromatograms of the *PRKD1* E710D hotspot mutation in cases of polymorphous adenocarcinoma of the salivary gland.

**Figure S3:** Correlation matrix between immunostaining for HH pathway proteins and PRKD1 in cases of polymorphous adenocarcinoma.

**Table S1:** Description of primary antibodies and reagents for immunohistochemistry. Note. OSCC: oral squamous cell carcinoma.

**Table S2:** Synthesis of studies reporting the frequency of the PRKD1 E710D mutation and associated histological patterns.

**Table S3:** Distribution of immunostaining of Hedgehog pathway proteins in cases of polymorphous adenocarcinoma of the salivary gland.

## Data Availability

The data that support the findings of this study are available on request from the corresponding author. The data are not publicly available due to privacy or ethical restrictions.
