# Supplementary figures and images for: Hedgehog Components Are Present in Polymorphous Adenocarcinoma of the Salivary Gland Regardless of PRKD1 Mutation and Tissue Invasion
Source: J Oral Pathol Med. 2025 Sep 10;54(10):1053–61. doi: 10.1111/jop.70057 (PMC12602139; doi:10.1111/jop.70057)

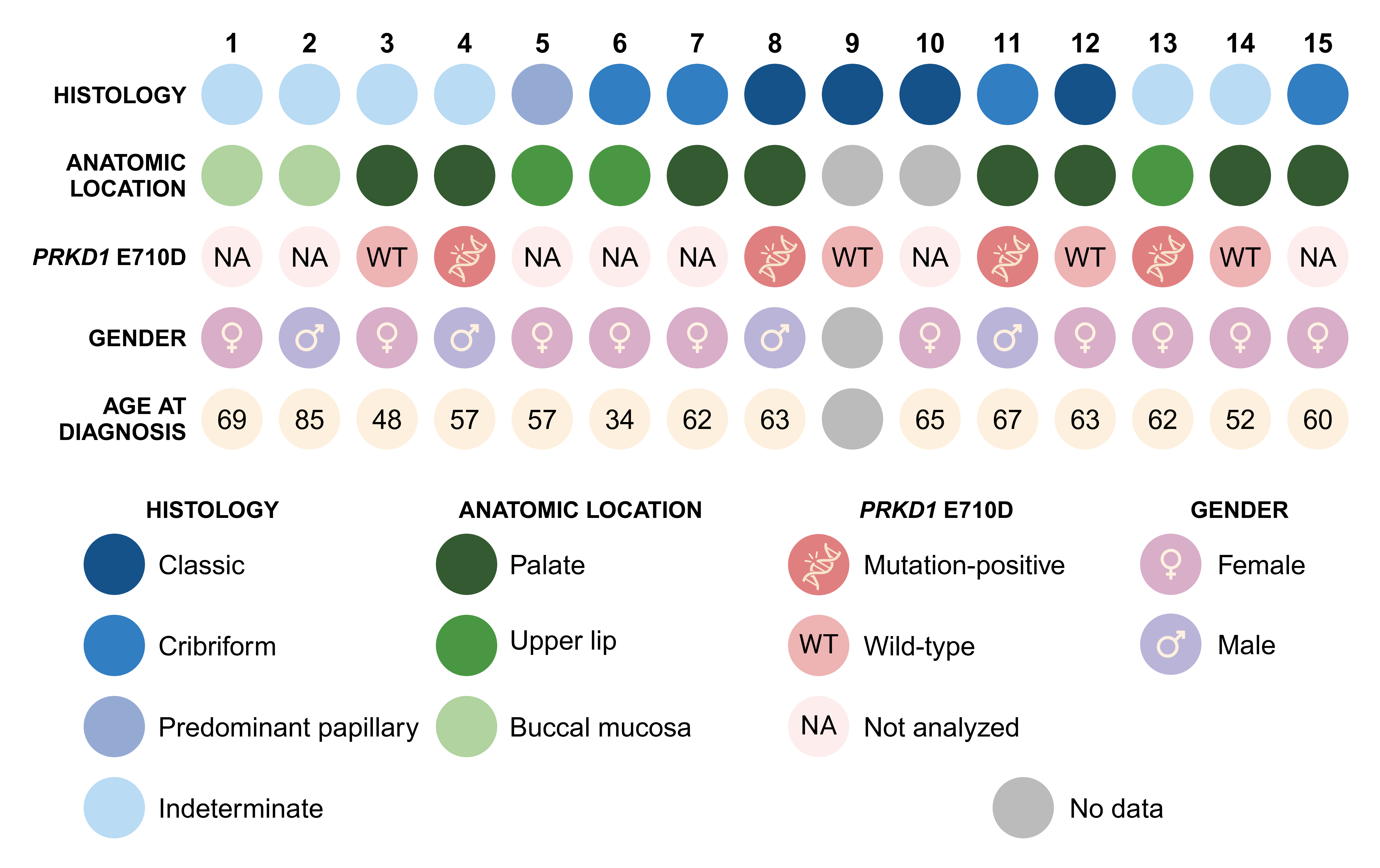

Supplement: Supplementary file 1 — Figure S1: Clinicopathological and demographic data and mutations of polymorphous adenocarcinomas of the salivary gland. [file JOP-54-1053-s002.tif]

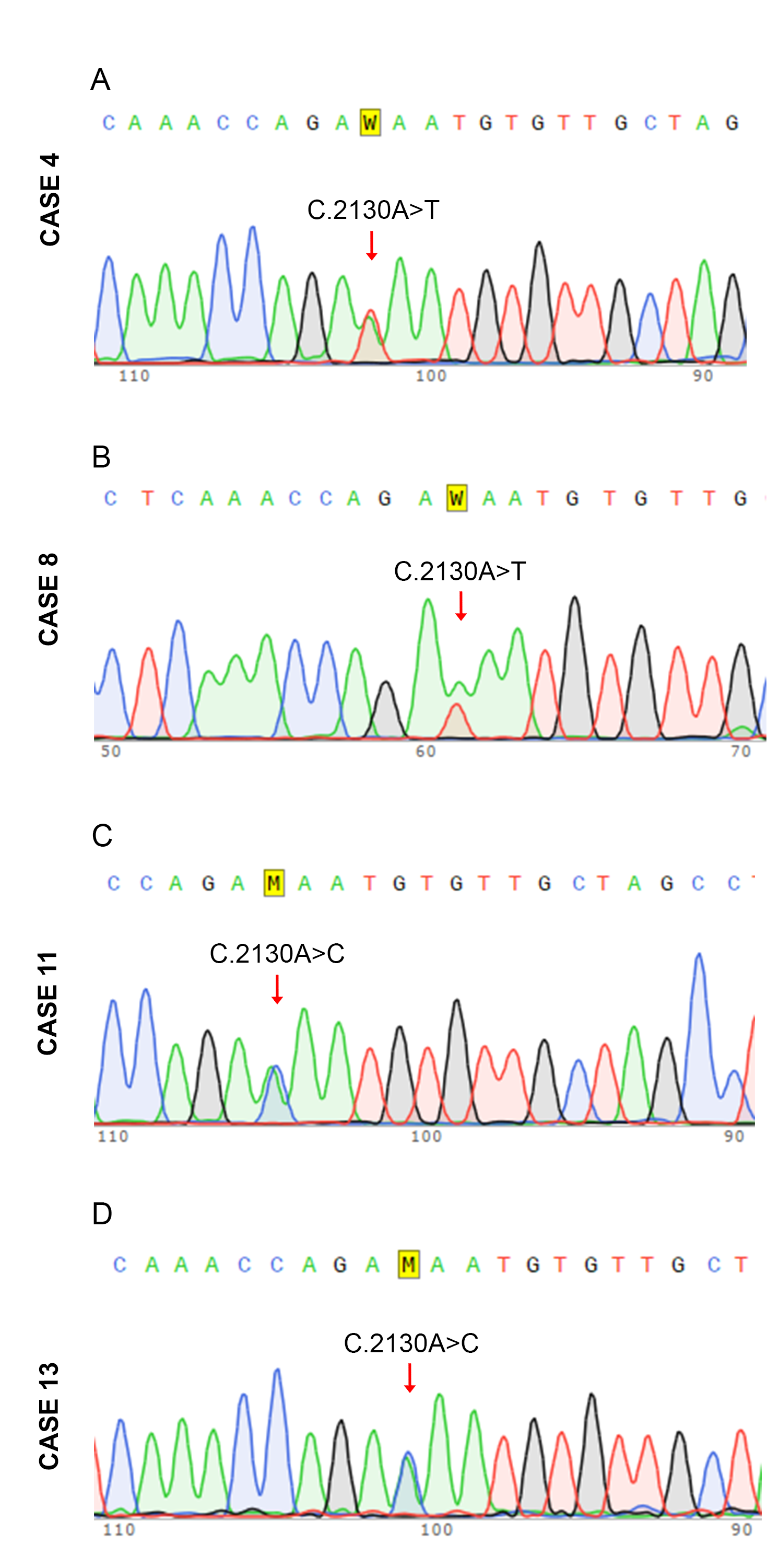

Supplement: Supplementary file 2 — Figure S2: Sanger sequencing chromatograms of the PRKD1 E710D hotspot mutation in cases of polymorphous adenocarcinoma of the salivary gland. [file JOP-54-1053-s003.tif]

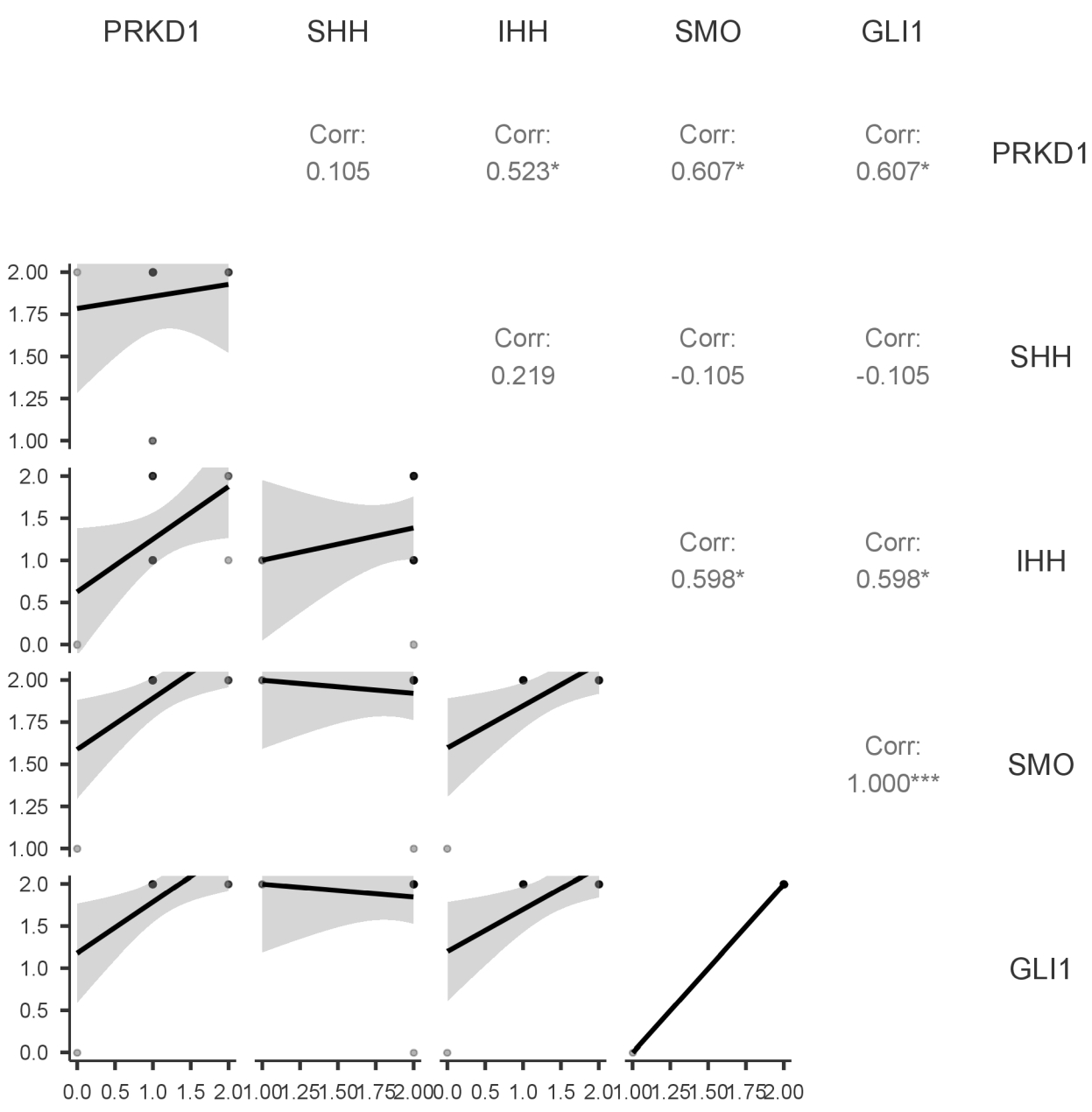

Supplement: Supplementary file 3 — Figure S3: Correlation matrix between immunostaining for HH pathway proteins and PRKD1 in cases of polymorphous adenocarcinoma. [file JOP-54-1053-s004.pdf]
