# Supplementary material for: Hedgehog Components Are Present in Polymorphous Adenocarcinoma of the Salivary Gland Regardless of PRKD1 Mutation and Tissue Invasion
Source: J Oral Pathol Med. 2025 Sep 10;54(10):1053–61. doi: 10.1111/jop.70057 (PMC12602139; doi:10.1111/jop.70057)
Supplement: Supplementary file 4 — Table S1: Description of primary antibodies and reagents for immunohistochemistry. Note. OSCC: oral squamous cell carcinoma. [file JOP-54-1053-s001.docx]

Supplementary Table 1. Description of primary antibodies and reagents for immunohistochemistry

| **Antibody** | **Manufacturer** | **Clone** | **Dilution** | **Antigen retrieval** | **Positive control** |
| --- | --- | --- | --- | --- | --- |
| PRKD1 | Merck | Polyclonal | 1:200 | EDTA | OSCC |
| SHH | Invitrogen | Monoclonal | 1:1,500 | EDTA | OSCC |
| IHH | Abcam | Monoclonal | 1:500 | EDTA | OSCC |
| GLI-1 | Invitrogen | Monoclonal | 1:600 | EDTA | OSCC |
| SMO | Merck | Polyclonal | 1:400 | EDTA | OSCC |

Note. OSCC: oral squamous cell carcinoma
