# Supplementary material for: Hedgehog Components Are Present in Polymorphous Adenocarcinoma of the Salivary Gland Regardless of PRKD1 Mutation and Tissue Invasion
Source: J Oral Pathol Med. 2025 Sep 10;54(10):1053–61. doi: 10.1111/jop.70057 (PMC12602139; doi:10.1111/jop.70057)
Supplement: Supplementary file 5 — Table S2: Synthesis of studies reporting the frequency of the PRKD1 E710D mutation and associated histological patterns. [file JOP-54-1053-s006.docx]

Supplementary Table 2. Synthesis of studies reporting on the frequency of the *PRKD1* E710D mutation and associated histological patterns

| Author (year) | n | Gender | Age (years) | Method | E710D mutation | Histological pattern in mutated cases |
| --- | --- | --- | --- | --- | --- | --- |
| Weinreb *et al*. (2014) | 59 | - | - | NGS and Sanger | 43/59 (72.9%) | - |
| Andreasen *et al*. (2018) | 18 | 7 M  11 F | 37-80  Mean 60 | Sanger | 9/18 (50%) | - |
| Sebastiao *et al*. (2019) | 3 | 2 M  1 F | 58-82  Mean 71 | NGS and Sanger | 2/3 (66,6%) | - |
| Sebastiao *et al*. (2020) | 37 | 14 M  23 F | 34-84  Mean 60 | Sanger | 13/37 (35%) | 5 classic  2 cribriform  6 indeterminate |
| Freiberguer *et al*. (2021) | 6 | 3 M  3 F | 33-84  Mean 57 | Sanger | 3/6 (50%) | 2 classic  1 cribriform |
| Clausen *et al*. (2022) | 31 |  | 13-87  Mean 59 | Sanger | 16/31 (51.6%) | - |
| Fukumura *et al*. (2022) | 36 | 20 M  16 F | 27-88  Mean 63 | Sanger | 4/36 (11.1%) | - |
| Hahn *et al*. (2023) | 51 | 12 M  39 F | 34-93  Mean 60 | NGS | 10/51 (19.6%) | 6 cribriform  4 mixed/indeterminate |
| Present study (2025) | 8 | 3 M  4 F | 34-85  Mean 60 | Sanger | 4/8 (50%) | 1 classic  1 cribriform  2 indeterminate |

Note. M: male; F: female; NGS: next-generation sequencing.
