# Supplementary material for: Hedgehog Components Are Present in Polymorphous Adenocarcinoma of the Salivary Gland Regardless of PRKD1 Mutation and Tissue Invasion
Source: J Oral Pathol Med. 2025 Sep 10;54(10):1053–61. doi: 10.1111/jop.70057 (PMC12602139; doi:10.1111/jop.70057)
Supplement: Supplementary file 6 — Table S3: Distribution of immunostaining of Hedgehog pathway proteins in cases of polymorphous adenocarcinoma of the salivary gland. [file JOP-54-1053-s005.docx]

Supplementary Table 3. Distribution of immunostaining of Hedgehog pathway proteins in cases of polymorphous adenocarcinoma of the salivary gland.

|  |  | **PRKD1**  n (%) | **SHH**  n (%) | **IHH**  n (%) | **SMO**  n (%) | **GLI-1**  n (%) |
| --- | --- | --- | --- | --- | --- | --- |
| **Cases** | Absent expression | 1 (6.7) | - | 1 (6.7) | - | 1 (6.7) |
|  | Low expression | 11 (73.3) | 2 (13.3) | 8 (53.3) | 1 (6.7) | - |
|  | High expression | 3 (20.0) | 13 (86.7) | 6 (40.0) | 14 (93.3) | 14 (93.3) |
| **Localization** | Absent | 1 (6.7) | - | 1 (6.7) | - | 1 (6.7) |
|  | Cytoplasmic | 12 (80.0) | 12 (80.0) | 5 (33.3) | 7 (46.7) | 1 (6.7) |
|  | Nuclear | 1 (6.7) | - | - | 3 (20.0) | 6 (40.0) |
|  | Cytoplasmic and nuclear | 1 (6.7) | 1 (6.7) | - | 4 (26.7) | 6 (40.0) |
|  | Membrane | - | 2 (13.3) | 7 (46.7) | 1 (6.7) | 1 (6.7) |
|  | Cytoplasmic and membrane | - | - | 2 (13.3) | - | - |
